# Enhancing *Saccharomyces cerevisiae* Taxane Biosynthesis and Overcoming Nutritional Stress-Induced Pseudohyphal Growth

**DOI:** 10.3390/microorganisms10010163

**Published:** 2022-01-13

**Authors:** Laura Ellen Walls, José L. Martinez, Leonardo Rios-Solis

**Affiliations:** 1 Institute for Bioengineering, School of Engineering, University of Edinburgh, Edinburgh EH9 3DW, UK; laura.walls@ed.ac.uk; 2 Centre for Synthetic and Systems Biology (SynthSys), University of Edinburgh, Edinburgh EH9 3BF, UK; 3 Department of Biotechnology and Biomedicine, Section for Synthetic Biology, Technical University of Denmark, 2800 Kongens Lyngby, Denmark; jlmr@dtu.dk

**Keywords:** *Saccharomyces cerevisiae*, Taxol, pseudohyphae, nutritional stress, bioprocess optimization, high-throughput microbioreactor, scale-up, taxadiene-5α-ol, taxadien-5α-yl acetate

## Abstract

The recent technological advancements in synthetic biology have demonstrated the extensive potential socio-economic benefits at laboratory scale. However, translations of such technologies to industrial scale fermentations remains a major bottleneck. The existence and lack of understanding of the major discrepancies in cultivation conditions between scales often leads to the selection of suboptimal bioprocessing conditions, crippling industrial scale productivity. In this study, strategic design of experiments approaches were coupled with state-of-the-art bioreactor tools to characterize and overcome nutritional stress for the enhanced production of precursors to the blockbuster chemotherapy drug, Taxol, in *S. cerevisiae* cell factories. The batch-to-batch variation in yeast extract composition was found to trigger nutritional stress at a mini-bioreactor scale, resulting in profound changes in cellular morphology and the inhibition of taxane production. The cells shifted from the typical budding morphology into striking pseudohyphal cells. Doubling initial yeast extract and peptone concentrations (2×YP) delayed filamentous growth, and taxane accumulation improved to 108 mg/L. Through coupling a statistical definitive screening design approach with the state-of-the-art high-throughput micro-bioreactors, the total taxane titers were improved a further two-fold, compared to the 2×YP culture, to 229 mg/L. Filamentous growth was absent in nutrient-limited microscale cultures, underlining the complex and multifactorial nature of yeast stress responses. Validation of the optimal microscale conditions in 1L bioreactors successfully alleviated nutritional stress and improved the titers to 387 mg/L. Production of the key Taxol precursor, T5αAc, was improved two-fold to 22 mg/L compared to previous maxima. The present study highlights the importance of following an interdisciplinary approach combining synthetic biology and bioprocessing technologies for effective process optimization and scale-up.

## 1. Introduction

**Figure 1:**
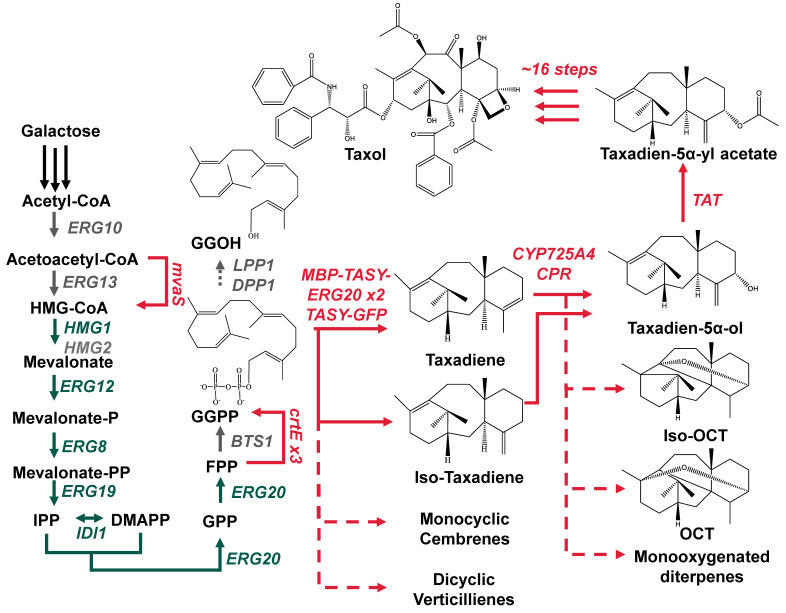
Engineered biosynthetic pathway in the Saccharomyces cerevisiae strain LRS6. Galactose is converted to the universal diterpenoid precursor, GGPP, via the mevalonate pathway. The first enzyme in the paclitaxel pathway, TASY, catalyzes the conversion of GGPP to taxadiene and small amounts of its isomer, iso-taxadiene. The second enzyme, CYP725A4, coupled with its cognate reductase subsequently catalyzes the oxidation of taxadiene to T5αol. The third enzyme, TAT, then catalyzes the acetylation of T5αol. The native, overexpressed genes are highlighted in green, whilst heterologous genes are highlighted in red. The dashed arrows highlight the undesirable side-products; the structures of the two well-characterized by-products, OCT and iso-OCT are shown.

Recent technological advancements within the field of synthetic biology [1,2,3,4] have facilitated the acceleration of efforts to develop an alternative, more sustainable source of diterpenoid-based pharmaceuticals, such as Taxol. Reconstruction of the early steps of the highly complex Taxol biosynthetic pathway in microbial cell factories has been achieved at laboratory scale [5,6,7,8] (Figure 1). However, low yields of the early taxadiene, T5αol and T5αAc intermediates remains a major bottleneck in biosynthetic pathway development. Work to reconstruct the pathway has revealed deviations in the microbial host, media composition and processing conditions can have a profound impact on the performance of early pathway enzymes [7,8,9]. Deviations in the medium composition, temperature and pH had a substantial impact on the product spectra and titers obtained in *S. cerevisiae* cultures [7,8,9]. Such findings are likely the result of a lack of understanding of the downstream effects associated with the heterologous overproduction of small molecules, such as those resulting from the expression of the early Taxol pathway. The overexpression of native and heterologous genes can induce changes in physiology, intracellular pH, redox balance and viability [10,11], and further understanding of the nature of these changes and their effect on stress tolerance is essential to ensuring robust performance under harsh industrial conditions.

Undefined complex materials, such as yeast extract and peptone, are often preferred for industrial bioprocesses, due to their relatively low cost and richer supply of nutrients compared to defined substrates [12,13]. However, lot-to-lot variations in the composition of yeast extract have been found to cause nutritional stress with a major impact on strain performance [13,14]. As the scale is increased during bioprocess scale-up, the mixing efficiency is reduced and spatiotemporal substrate, oxygen, pH and temperature gradients develop across the reactor volume [15,16], exacerbating such effects. *S. cerevisiae* makes use of complex signal transduction pathways to adapt to changes in the availability of essential nutrients [17]. Such pathways stimulate proliferation under nutrient-rich conditions and induce entry into a quiescent “stationary phase” under nutrient scarcity [17]. For example, upon the depletion of glucose, the Crabtree-positive yeast, *S. cerevisiae* has evolved to enter a short stationary phase, whilst it reprograms its metabolism for growth on ethanol. Another interesting characteristic response to nutrient stress in many fungal species, including *S. cerevisiae*, is filamentous growth [18]. In diploid cells, nitrogen starvation triggers a profound change in cellular morphology, from normal budding yeast form to elongated pseudohyphal cells, which are separated by cytokinesis but attached through adhesion proteins [19]. In haploid cells, a similar but distinct phenomenon, termed invasive growth, occurs in response to glucose starvation [19]. This elongated growth has been predominantly reported on solid nutrient-limited agar cultures and is believed to be a foraging mechanism to scavenge for nutrients when availability is scarce [20]. Interestingly, the presence of quorum-sensing molecules in the cultivation medium has been found to elicit a similar response, even in liquid media [21]. Quorum sensing is a means of cell-to-cell microbial communication and the detection of quorum sensing molecules has been found to trigger profound changes in gene expression in a wide range of microbial species [22,23]. Fungal quorum sensing was first discovered in *C. albicans* when the sesquiterpene alcohol, farnesol, was found to inhibit the yeast to hyphal switch in the species [24]. In *S. cerevisiae*, fusel alcohols, including tryptophol and phenylethanol, have been found to be quorum-sensing molecules [22]. The secretion of tryptophol and phenylethanol is tightly controlled by cell density and is subject to positive feedback [22]. The expression of key genes involved in amino acid metabolism, aromatic amino acid aminotransferase 2 (*ARO9*) and transaminated amino acid decarboxylase (*ARO10*) was upregulated up to around 50-fold in high density (5 × 10^7^ cells/mL), compared to low density (10^5^ cells/mL) cultures [22]. The resulting aromatic alcohol products induce the expression of the *FLO11* gene [22], the product of which is a cell surface flocculin that is vital for filament formation in the strain. The production of these aromatic alcohols is further regulated by nitrogen, and the pathway is repressed by ammonia and activated under nitrogen-poor conditions.

The limited insight and understanding of the process during preliminary screening, and the failure to account for discrepancies in critical cultivation conditions, such as nutrient availability, can cause major scale-up challenges [25]. Detailed process characterization in these early stages of bioprocess development is therefore crucial to understand how such factors can affect the adaptation of optimization strategies to large-scale bioreactor cultivations [16,26]. In this study, a statistical definitive screening design (DSD) approach is coupled with state-of-the-art micro and mini bioreactor technologies with the aim of developing a robust, scalable bioprocess for heterologous taxane production. A particular emphasis was made on overcoming the nutritional stress-induced filamentous growth in engineered *S cerevisiae* cells to improve process scalability and reliability. The statistical relationships between factors and performance were determined in line with quality by design principles. The optimal conditions elucidated at a microscale were validated using highly instrumented benchtop bioreactors.

## 2. Materials and Methods

### 2.1. Yeast Strains

The yeast strains used in this study were LRS6 (MATa, leu2-3, 112::HIS3MX6-GAL1p-ERG19/GAL10p-ERG8;ura3-52::URA3-GAL1p-MvaSA110G/GAL10p-MvaE (codon optimized); his3Δ1::hphMX4-GAL1p-ERG12/GAL10p- IDI1; trp1-289::TRP1_GAL1p-CrtE(X.den)/GAL10p-ERG20;YPRCdelta15::NatMX-GAL1p-CrtE(opt)/GAL10p-CrtE; ARS1014::GAL1p-TASY-GFP; ARS1622b::GAL1p-MBP-TASY-ERG20; ARS1114a::TDH3p-MBP-TASY-ERG20; ARS511b::GAL1p-CYP725A4-PGK1t/GAL3p-CPR-ENO2t; RKC3::GAL1p-TAT-CYC1t), as previously described [7,8], originating from CEN.PK2-1C (EUROSCARF collection).

### 2.2. MiniBio 500 Bioreactor Cultivation

The cultivations were conducted in MiniBio 500 bioreactors (Applikon Biotechnology, Delft, The Netherlands) with a working volume of 250 mL. Pre-inoculum cultures were prepared by transferring from a single colony to 5 mL of YPD (yeast extract 10 g/L; peptone 20 g/L, glucose 20 g/L) and incubating at 30 °C and 250 rpm for 8 h. The resulting culture was subsequently used to inoculate a secondary culture to an OD_600_ = 1 and incubated overnight. An aliquot of the resulting culture was diluted with YPG (yeast extract 10 g/L, peptone 20 g/L, galactose 20 g/L) to give a 200 mL culture with an initial OD_600_ = 1. In the 2×P culture, the concentration of peptone was doubled to 40 g/L, whilst in the 2×YP cultivation both the yeast extract and peptone concentrations were doubled to 20 and 40 g/L, respectively. 

To prevent excess foam production, polypropylene glycol P2000 (Alfa Aesar, Loughborough, UK) was added to a concentration of 0.01% (*v/v*) and a Rushton turbine was placed at the medium–air interface. A 20% dodecane overlay was also added. The temperature, DO and pH were measured online. The adaptive my-control system (Applikon Biotechnology, Delft, The Netherlands) was used to control the DO and temperature to setpoints of 30% saturation and 30 °C, respectively. Medium pH was maintained above 6 through the automatic addition of 1 M NaOH. Where the biomass was measured online, a BE2100 OD scanner (BugLab, Concord, MA, USA) was employed, and offline measurements were performed twice daily using a Nanodrop 2000c spectrophotometer (Thermo Fisher Scientific, Loughborough, UK). Samples were taken twice daily for the analysis of taxanes via GC-MS and galactose using the DNS method [27]. All the chemicals were purchased from Fisher Scientific, UK, at the highest available purity, unless otherwise stated.

### 2.3. Microscale Complex Media Optimization

The effect of six key factors, initial OD_600_, yeast extract lot along with yeast extract, peptone, galactose and additional uracil concentrations on the total taxane and biomass accumulation was investigated. A definitive screening design (DSD) was created using JMP Pro 15 statistical software, as summarized in Table S1.

Inoculum cultures were prepared by transferring a single colony of *LRS6* to 5 mL of YPD media and incubating at 30 °C and 250 rpm overnight. Aliquots of the inoculum cultures were diluted with each of the 14 media to give an 800 μL culture with the desired initial OD_600_, as indicated in Table S1. A 200 µL dodecane overlay was also added to each well, to prevent the loss of the volatile taxane products due to air stripping, giving a total working volume of 1 mL. The temperature was maintained at 30 °C under an agitation of 1000 rpm with a shaking diameter of 3 mm in 48-well Flowerplates (mp2-labs, Beasweiler, Germany). The temperature, biomass, dissolved oxygen (DO) and pH were monitored online using the inbuilt optical sensors. Taxane production was analyzed via GC-MS at the end of the cultivation.

### 2.4. Bioreactor Cultivation

The optimal conditions from the microscale optimization study were validated using 1L BIOSTAT Q plus bioreactors (Sartorius-Stedim Biotech S.A, Goettingen, Germany), with a working volume of 500 mL. Pre-inoculum cultures were prepared by transferring a single *LRS6* colony to 5 mL of YPD and incubating at 30 °C and 250 rpm overnight. The resulting cultures were subsequently diluted with 45 mL of fresh YPD medium in 500 mL Erlenmeyer flasks and incubated at 30 °C for 24 h. An aliquot of the resulting culture was diluted with the optimized media to give a 400 mL culture with an initial OD_600_ = 0.79. Antifoam 204 (Sigma-Aldrich, Goettingen, Germany) was added as needed to control excess foam production. A 100 mL dodecane overlay equivalent to 20% of the total volume was also added. Temperature, DO and pH were measured online. MFCS software (Version 3.0, Sartorius-Stedim Biotech S.A, Goettingen, Germany) was employed to control the BIOSTAT cultivation, pH was maintained at 6 through the automatic addition of 2 M NaOH or 2 M H_2_SO_4_ and the temperature was maintained at 30 °C. A constant airflow of 1 vvm was maintained and the stirrer speed was adjusted manually to maintain the dissolved oxygen above 30%. Off-gas analysis was performed online via mass spectrometry (Prima Pro, Thermo Fisher Scientific, Loughborough, UK). Samples were taken twice daily for taxane and metabolite quantification via GC-MS and HPLC, respectively.

### 2.5. Taxane and Metabolite Identification and Quantification

Taxane identification and quantification was achieved via GC-MS. The organic dodecane layer was separated from the culture medium through centrifugation, and a 1 μL sample was injected into a TRACE™ 1300 Gas Chromatograph (Thermo Fisher Scientific, Loughborough, UK) coupled to an ISQ LT single quadrupole mass spectrometer (Thermo Fisher Scientific, Loughborough, UK). Chromatographic separation was achieved using a Trace Gold TG-SQC gas chromatography column using a previously described method [28]. To identify and quantify the production of compounds by *LRS6,* standards of taxadiene, kindly supplied by Baran Lab (The Scripps Research Institute, La Jolla, California, CA, USA), and GGOH obtained from Sigma-Aldrich (Gillingham, UK) were used. Additional product concentrations were estimated relative to the standard taxadiene concentrations. In the BIOSTAT cultivation, ethanol, acetate and glycerol production were analyzed via ion-exchange HPLC. Following filtration using a 0.45 μm filter, 20 μL samples were injected into a Bio-Rad Aminex HPX-87H column (Hercules, CA, USA) for analysis. The eluent was 5 mM H_2_SO_4_, the flowrate was 0.6 mL/min and the temperature was 60 °C. An RID detector was used for quantification.

### 2.6. Statistical Analysis

The design of experiments and statistical analyses were performed using JMP Pro 15 statistical software. A DSD with a two-level categorical factor was used [29]. Statistical model development involved the use of forward stepwise regression in JMP Pro 15 using the combine option and a *p*-value of 0.1, and all terms up to the second order were considered [29]. The null hypothesis considered that there was no significant difference between the cultures; hence, if *p* ≤ 0.05, the null hypothesis was rejected.

## 3. Results and Discussion

### 3.1. Reproducibility and Factor Determination

**Figure 2:**
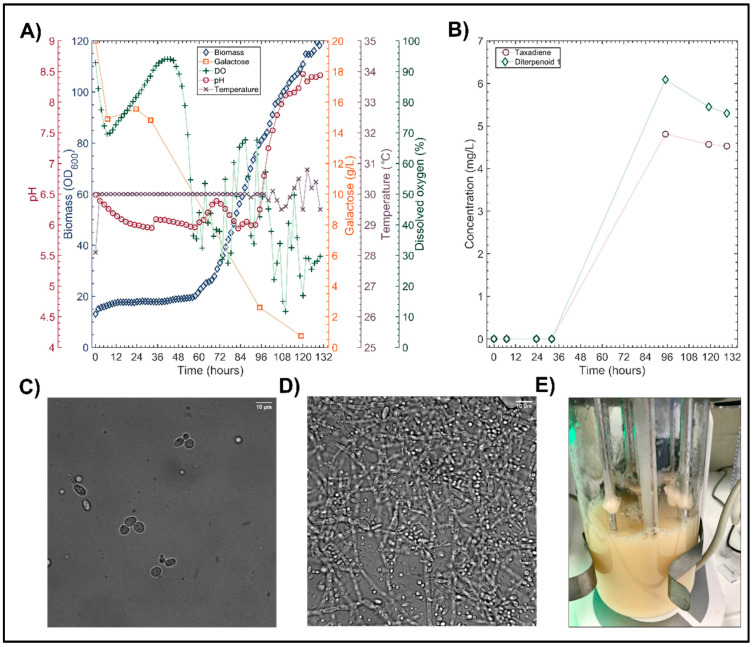
MiniBio 500 bioreactor cultivation. LRS6 was cultivated in the Applikon Minibio 500 bioreactors in YPG prepared with yeast extract lot 175915. (**A**) The temperature (×), pH (ο) and dissolved oxygen (+) were monitored and controlled online to the set points of 30 °C, 6 and 30%, respectively. The biomass (⋄) was monitored online using a BE2100 OD scanner (BugLab, Concord, CA, USA). The galactose consumption (□) was monitored offline via sampling twice daily. (**B**) Total taxane (o) and diterpenoid 1 (⋄) accumulation was measured via the GCMS analysis of dodecane extracts of offline samples. Oil immersion brightfield microscope images taken at 32 h (**C**) and at the end of the cultivation (**D**) using a 100× lens. (**E**) Biofilm formation observed on the bioreactor probes.

The lack of reproducibility within the life sciences has recently been deemed a “crisis” in the biological sciences; in particular, over 77% of research scientists admitted to having tried and failed to repeat another’s laboratory experiment in a recent survey [30,31]. This greatly hinders bioprocess development and the translation of biotechnological advancements to industrial processes. In order to assess process repeatability and provide a starting point for the optimization in this study, a previous *LRS6* cultivation [8] was repeated. The results of this experiment are summarized in Figure 2.

When the *LRS6* YPG cultivation was repeated, the final OD_600_ value increased 3.5-fold to 119.6, compared to the previous cultivation under the same conditions [8], as shown in Figure 2A. A significant amount of biofilm formation was observed on the surfaces of the probes and agitator within the bioreactor, as shown in Figure 2E. This unusual growth was also highly detrimental to heterologous gene expression, as the maximum total taxane titer was five-fold lower than the previous cultivation [8], at just 10.9 mg/L (Figure 2B). The visualization of the cells using oil immersion bight field microscopy at the end of the cultivation, revealed a filamentous morphology (Figure 2D). As the visualization of the cells at 32 h (Figure 2C) revealed the expected budding morphology, the observed morphological aberrations were likely a response to the stress within the bioreactor. Biomass was measured as absorbance at 600 nm, and was therefore inherently sensitive to such changes in the cellular morphology. As the abnormally high OD_600_ readings observed in Figure 2A occurred in the final 48 h, at which point over 80% of the primary carbon source had been depleted, they are likely the result of interference due to cellular elongation.

### 3.2. Effect of the Medium on Pseudohyphal Growth in the Minibio 500 Reactor

Filamentous growth is a characteristic response to nutrient stress in *S. cerevisiae*; in haploid cells it is commonly the result of glucose starvation, whilst nitrogen starvation is often the cause in diploid cells [32]. As the parent yeast strain used in this study was a haploid uracil auxotroph derived from CEN.PK2-1C, glucose starvation could be a potential cause of the observed filamentous growth. However, although sub-optimal carbon sources, including galactose and ethanol, have been shown to enhance filamentous growth [32], filamentous growth was not observed in the previous cultivations [8]. In addition, the previous cultivation of the strains at microscale, in YP media supplemented with 0.2% sugar that was ten-fold lower than the 2% sugar added in the bioreactor cultivations, revealed no signs of filamentous morphology [8]. The nature and concentration of the carbon source was therefore unlikely to be the sole cause of the filamentous morphology observed.

**Figure 3:**
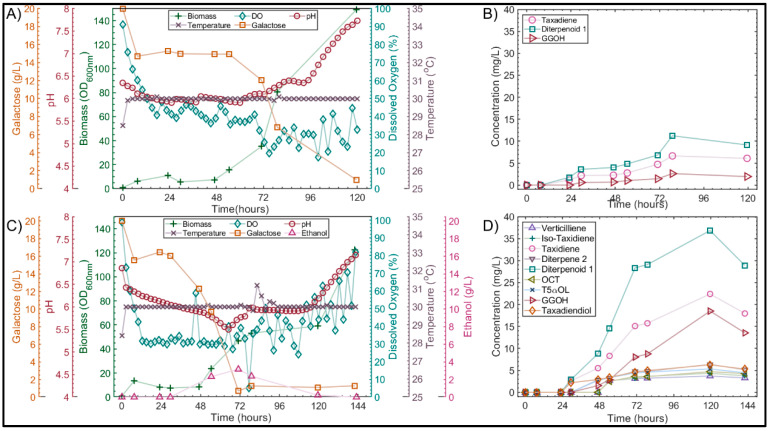
The 2×P and 2×YP bioreactor cultivation results. The initial concentration of peptone (2×P) and both yeast extract and peptone (2×YP) in the YPG media were doubled in *LRS6* cultivations. Temperature (×), pH (o) and dissolved oxygen (⋄) were monitored and controlled online. Biomass (+), galactose (□) and ethanol (Δ) concentration were monitored via offline sampling twice daily for 2×P (**A**) and 2×YP (**C**) cultivations. The taxane concentration was measured via the GCMS analysis of dodecane samples taken twice daily for 2×P (**B**) and 2×YP (**D**).

Nitrogen limitation has been commonly reported to induce filamentous growth in diploid *S. cerevisiae* cells [20]. Although the cells in this study were haploid, an additional cultivation was performed in which the concentration of the main nitrogen source (peptone) was doubled to rule out nitrogen limitation as the cause of the nutritional stress response observed in this study, as shown in Figure 3A,B.

**Figure 4:**
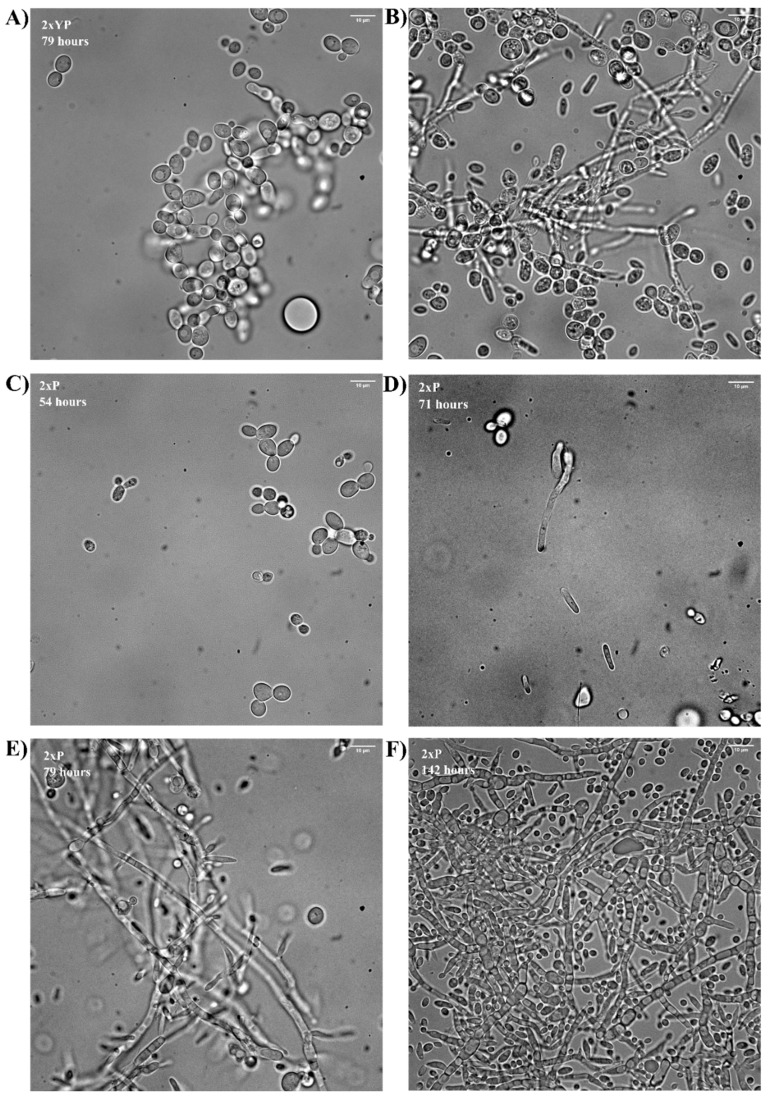
Microscope images taken during 2×YP and 2×P cultivation. Brightfield images were taken using a 100× oil immersion lens. Image taken from the 2×YP cultivation at 79 (**A**) and 119 (**B**) hours. Images taken of the samples taken from the 2×P cultivation at 54 (**C**), 71 (**D**), 79 (**E**) and 142 (**F**) hours, respectively.

Cellular morphology was monitored throughout the cultivations via oil immersion bright field microscopy, as summarized in Figure 4. In the 2×P cultivation, the cells were mostly in the budding yeast form in the sample taken at 54 h (Figure 4C); however, by 71 h, some elongation of the cells could be observed (Figure 4D). At this point, over half of the primary carbon source was still available (Figure 3A). By 77 h, substantial pseudohyphae formation was observed (Figure 4E) and the cells remained in the filamentous formation for the remainder of the cultivation (Figure 4F). Taxane production was detected through GC-MS analysis of the dodecane extracts (Figure 3B); however, the maximum total taxane titer was 20 mg/L, almost three-fold lower than that achieved in the previous YPG cultivation [8].

Although all cultivation conditions were kept the same as in the previous cultivation, the use of a fresh lot of yeast extract (Fisher Scientific Loughborough, UK, lot: 175915) was necessary to prepare the YPG media. Yeast extract commonly forms a major component of industrial fermentations [13]; however, lot-to-lot variations in its composition can have a major impact on productivity. Such variations resulted in differences of up to 50% in the accumulation of biomass [14]. Previous work revealed a strong correlation between biomass and taxane accumulation in the *S. cerevisiae* strain, *LRS6* [8]. It was therefore hypothesized that the variations in the yeast extract composition could have contributed to the deviations in the performance observed. A further scoping cultivation was therefore performed, in which the concentration of both the yeast extract (Fisher Scientific, Loughborough, UK, lot: 175915) and peptone were doubled. The results of this experiment are summarized in Figure 3C,D.

Interestingly, when both the initial yeast extract and peptone concentrations were doubled, the total taxane titer improved 5.4-fold to 108 mg/L, as shown in Figure 3D. The cells displayed the expected budding morphology in the samples taken between 0 and 77 h (Figure 4A). Morphological aberrations were observed in the final 48 h of the culture (Figure 4B); however, in contrast to the previous cultivations (Figure 2 and Figure 3B), this did not inhibit the taxane titers. The maximum titer of the major oxygenated taxane compound, diterpenoid 1 and the critical Taxol intermediates taxadiene and T5αol, were 37, 22 and 5 mg/L (Figure 3D), respectively, 1.9-, 2.2- and 1.6-fold higher than those achieved in the controlled cultivation with the original yeast extract lot [8]. Ethanol production was monitored during this experiment, as shown in Figure 3C. Aerobic fermentation of the galactose occurred between 29 and 71 h with a maximum ethanol titer of 3.2 g/L being reached. After 71 h, the majority of the galactose had been consumed and growth proceeded via aerobic ethanol metabolism. Upon the depletion of the two main carbon sources, a substantial increase in pH and a morphologic switch was observed. An increase in the extracellular pH in the latter stages of cultivation has been associated with the release of highly basic ammonia during amino acid catabolism [33]. Upon the depletion of more preferable nitrogen sources, such as ammonium and glutamine in the cultivation medium, *S. cerevisiae* will catabolize the less preferable branched chain and aromatic amino acids producing fusel alcohols as by-products [34]. Certain fusel alcohols are known as quorum-sensing molecules in *S. cerevisiae*, and have an important role in the induction of genes, such as *FLO11*, which are responsible for morphogenesis in the species [22]. It was therefore hypothesized that the accumulation of certain quorum-sensing molecules could have played a role in the morphological changes observed.

### 3.3. Quorum Sensing in S. cerevisiae

The nitrogen source used in this investigation was peptone from casein (pancreatic digest), which is a rich source of tryptophan with a 2.7-fold higher content than that from the enzymatic digest of animal protein [35]. Interestingly, the quorum-sensing molecule tryptophol, which promotes filamentous growth, is the product of the assimilation of tryptophan via the Ehrlich pathway [36]. In *C. albicans* cultures, supplementation of the media with tryptophan was found to increase tryptophol biosynthesis 2.5-fold [37]. The use of casein peptone could have therefore enhanced tryptophol production in the cultivations performed in this study, and contributed to the unusual growth observed; however, as no evidence of high level accumulations of these compounds was detected in the solvent extracts, further investigation is needed to confirm this.

The detailed investigation of the dodecane extracts of the bioreactor samples revealed the accumulation of several compounds exclusively during filamentous growth, as summarized in Figure S1. For the 2×P cultivation, the chromatograph was similar to that of the solvent blank (Figure S1A) at 71 h, at the retention times between 3 and 5 min; however, an additional peak was observed at 79 h, at 3.55 min (Figure S1D). This compound showed a high degree of similarity to 5-dodecanol in the NIST mass spectral library. By 119.5 h, additional peaks at 3.38, 3.63 and 3.66 min can also be seen (Figure S1E). Through comparison of the corresponding mass spectra, the peaks were identified by the NIST mass spectral library as 5-dodecaone (72% probability), 2-dodecanone (85% probability) and 3-dodecanol (61% probability), respectively. Interestingly, the twelve-carbon alcohol, dodecanol, has been found to influence cellular morphology in *Candida albicans*, with a potent inhibitory effect on filamentous growth through a mechanism involving the transcription factor, *SFL1* [38]. A similar effect was observed when *C. albicans* was cultivated; in the presence of phenylethanol [22] and isoamyl alcohol [39], filamentous growth was inhibited. This quorum-sensing effect is opposite to that observed in *S. cerevisiae* [22,39]. According to recent reports, many alcohols, including dodecanol, have opposing quorum-sensing effects in *S. cerevisiae*, compared to *C. albicans* [39]. The exclusive detection of the dodecanol and dodecanone compounds in samples taken from *LRS6* cultivations demonstrating pseudohyphal growth, suggests that they could have had a quorum-sensing effect in the cultivations of this study. However, it is worth noting the high similarity of the compounds to the biocompatible solvent, dodecane, which was employed for the in situ liquid–liquid extraction of the taxane products. It is therefore difficult to determine whether the compounds were secreted by the yeast or are the result of the reaction of other compounds released by the filamentous cells with the dodecane overlay, and further study is needed to confirm this.

The filamentous growth of the *S. cerevisiae* strain *LRS6* observed in the MiniBio 500 cultivations was highly unusual. Previous studies have found that the growth of *LRS6* was reduced, compared to the *LRS5* strain, which was genetically identical to *LRS6*, except that it did not possess the *CYP725A4*, *CPR* and *TAT* genes responsible for the second and third steps in the Taxol biosynthetic pathway. This suggested that the integration of additional genes resulted in a decreased cellular fitness. In addition, the *LRS5* strain was recently found to have a reduced mechanical stress tolerance compared to the wild-type, CEN.PK-1C, strain [40]. In the presence of an in situ solid phase adsorbent resin with a concentration of 12% *w*/*v*, the growth of *LRS5* was completely inhibited, whilst the growth of the parental CEN.PK-1C was not significantly affected, further suggesting that metabolic engineering has a detrimental effect on cellular fitness and stress tolerance. The unusual stress response observed in this study was therefore likely to have been exacerbated by the reduction in fitness resulting from metabolic engineering.

### 3.4. Complex Media Optimization

#### 3.4.1. Microscale Media Optimization Using a Definitive Screening Design

**Figure 5:**
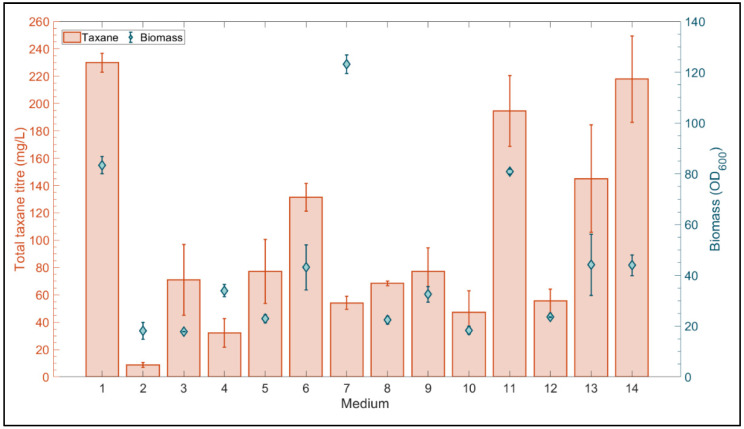
Microscale complex media screening results. Final total taxane and biomass titers obtained with each of the 14 media combinations in the BioLector. The values are mean ± standard deviation for triplicate cultivations.

The miniBio 500 cultivation results (Figure 2 and Figure 3) indicate that the composition and source of the nutrients in the cultivation broth had a major effect on both cellular morphology and taxane production by *LRS6*. As a result, a design of experiments guided, high throughput microscale optimization study was performed to screen a wider range of media compositions. An efficient three-level definitive screening design (DSD) was selected for this study, as this class of experimental design is capable of estimating the main effects without confounding with second order effects in just 2N + 1 runs for N factors. Unlike traditional resolution IV designs, each second order effect is not completely aliased with any other second order effect. In addition, the designs with 6–12 factors were found to be capable of the efficient estimation of all possible full quadratic models involving ≤3 active factors [41]. This has the potential to dramatically reduce time and resource requirements, as it is possible to progress to optimization without further experimentation. A DSD (Table S1) was created to investigate the effect of six key factors on taxane production by *LRS6*. Based on the initial experiments, the yeast extract lot, along with the concentrations of galactose, yeast extract and peptone, were deemed important factors. A strong positive correlation between biomass and taxane accumulation has also been observed for this strain [8], and the initial biomass concentration was therefore considered. Finally, as *LRS6* is a uracil auxotroph and the composition of industrially manufactured yeast extract has been found to be susceptible to deviations in nucleobase availability [13], it was hypothesized that additional uracil supplementation could help alleviate nutritional stress. A DSD was created using JMP Pro 15 (Table S1), to investigate the effect of these six factors on taxane production, the results of this experiment are summarized in Figure 5.

**Figure 6:**
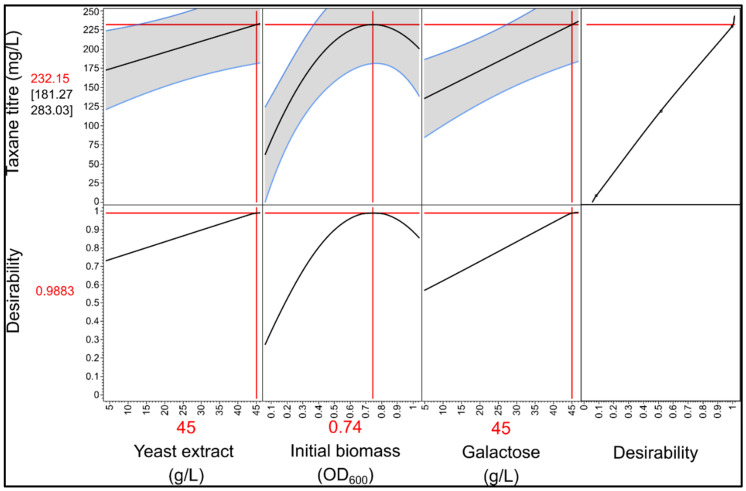
Optimization of taxane production using statistical modeling. The prediction profiler in JMP Pro 15 was used to determine the optimal factor combination according to the statistical model.

Of the 14 media combinations tested, medium one presented the highest total taxane titer of 229 ± 6 mg/L. Media combinations 11 and 14 also produced high taxane titers of 195 ± 26 and 218 ± 32 mg/L, respectively. Forward stepwise linear regression was performed using JMP Pro 15 statistical software with a *p*-value to enter of 0.1. A full quadratic analysis was performed, thereby considering all the main effects and any second order interactions. This revealed that initial OD_600_ (*p* = 0.011) along with yeast extract (*p* = 0.028) and galactose concentrations (*p* = 0.011) were the significant main effects. The non-linearity of the effect of initial OD_600_ (*p* = 0.007) was also elucidated, a major advantage of using a three-level screening design. According to the statistical model, the optimal initial yeast extract and galactose concentration was 45 g/L, and initial OD_600_ had an optimal value of around 0.74 (Figure 6). Increasing the concentration of peptone, and additional uracil supplementation resulted in no significant improvement to the taxane titer. Interestingly, no significant difference in the performance was observed due to the deviations in the yeast extract lot at this scale. At the end of the cultivations, the cells were visualized using oil immersion light microscopy and none of the cells exhibited filamentous morphology, indicating that the phenomenon was not solely due to the initial nutrient composition of the medium. In addition, none of the dodecanol or dodecanone compounds were detected in the dodecane extracts.

This experiment was conducted using the microscale bioreactor (BioLector Flowerplates), the unique design of which effectively overcomes oxygen limitation in *LRS6* cultivations by introducing a baffle-like structure [42]. Although the dissolved oxygen was controlled above 30% in the miniBio 500 reactor through automatic air sparging, oxygen availability was likely reduced compared to the FlowerPlate, in which it remained above 80% throughout all cultivations (Figure S2). *S. cerevisiae* is known to adapt to external oxygen availability by altering the amount of flux through respiratory and fermentation pathways [43] at a lower oxygen availability flux through respiratory pathways, and, hence, biomass accumulation is reduced and fermentation is increased [43]. In the original miniBio 500 cultivation, an OD_600_ of 21 was reached after 72 h of cultivation, significantly lower than the 37 ± 4 achieved in the equivalent microscale cultivation at 72 h [8]. Taxane accumulation was also reduced 1.5-fold at the larger scale [8]. Decreasing the rate of airflow from 1 vvm to 0.5 vvm or 0.25 vvm in the bioreactor cultivations was also found to reduce the terpenoid titer, 1.4-fold and 2.2-fold, in another *S. cerevisiae* strain [44]. In addition, the production of higher alcohols, including isobutanol and 3-methyl-butanol, which are known to induce filamentous growth [45], was found to have doubled in cultivations grown under anaerobic conditions, compared to those with higher dissolved oxygen tension [46]. Reduced oxygen availability at an increased scale may have therefore played a role in the observed response.

#### 3.4.2. Validation of Optimized Conditions in Highly Instrumented 1L Bioreactors

**Figure 7:**
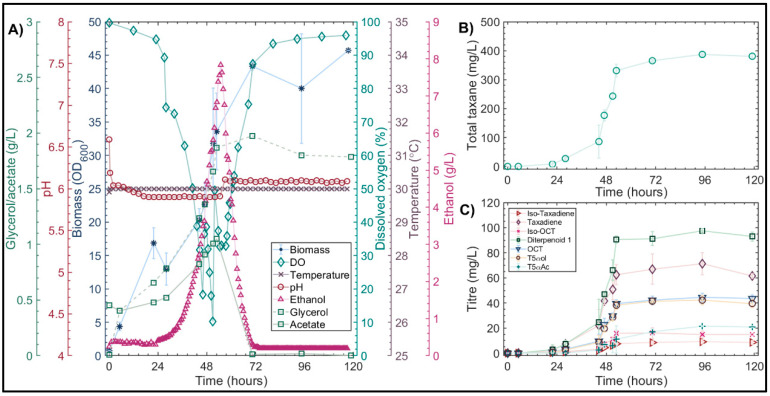
Optimized complex media in 1L BIOSTAT Bioreactor at 30 °C. (**A**) Biomass (*), ethanol (Δ), DO (⋄), temperature (×), pH (ο), acetate (–□) and glycerol (--□) production. (**B**) Total taxane production. (**C**) Accumulation of key Taxol intermediates. Biomass and taxane values are the mean ± standard deviation for two bioreactor cultivations. Other parameter values are from one replicate only.

In order to validate the optimized complex media elucidated at microscale, *LRS6* was cultivated in larger scale 1 L bioreactors. As it was hypothesized that reduced oxygen availability may have played a role in the stress response observed in previous bioreactor cultivations (Figure 2 and Figure 3), a constant airflow of 1 vvm was applied at this scale to minimize oxygen limitation. The results of this experiment are summarized in Figure 7.

During the first 21 h, an initial growth phase was observed in which the OD_600_ increased from 0.7 to 15.2 (Figure 7A). This was not coupled with ethanol production, and the respiratory quotient (RQ) was close to unity at 1.2. This may have therefore been due to the aerobic respiration of more favorable carbon sources present in the complex medium used. Respiratory-fermentative catabolism of the galactose occurred between 30 and 50 h, with a maximum ethanol concentration of 7.83 g/L. Acetate and glycerol were also produced with the maximum concentrations of 1.05 and 1.97 g/L, respectively. Following the depletion of galactose, the aerobic respiration of ethanol and acetate occurred between 50 and 72 h. At 72 h, all metabolizable carbon sources had been consumed and the stationary phase of growth was reached with an OD_600_ of 43.8.

In the GC-MS extracts, 10 diterpene products resulting from the heterologous expression of TASY were detected. The main diterpene product was taxadiene, with a maximum titer of 71 ± 8 mg/L observed at a cultivation time of 95 h (Figure 7B). The structural isomer, Iso-taxadiene [47], the known side-product verticilliene [47], and previously observed diterpene 1 [7,8], were also detected with maximum titers of 9 ± 1.7, 11 ± 0.1 and 12 ± 1 mg/L, respectively. An additional 6 compounds with a molecular ion peak of m/z = 272 were detected. A further 12 diterpenoid products of the CYP725A4 and TAT enzymes were observed. The major CYP725A4 product was the previously identified potential T5αol isomer, diterpenoid 1 [8,48], with a maximum titer of 97 ± 2 mg/L; Iso-OCT, OCT and T5αol were also detected with titers of 16 ± 3, 44 ± 3 and 42 ± 4 mg/L, respectively. The desired TAT product, T5αAc, was detected with a maximum titer of 21 ± 0.3 mg/L, representing an almost 6-fold improvement, compared to the previous literature [8]. An additional compound with a molecular ion peak of m/z = 304, equivalent to taxadiene (M_r_ = 272 g/mol) plus O_2_ (M_r_ = 32 g/mol), and a base peak of m/z = 245, was also produced with a maximum titer of 11 ± 5 mg/L. The mass spectrum of this compound was similar to that of a taxadiendiol that was produced as a result of the heterologous expression of CYP725A4 in *E. coli* [6]. An additional six unknown diterpenoid products were also detected. The total taxane titer was 387 ± 6 mg/L, representing a 1.7-fold improvement compared with that predicted by the statistical model (Figure 6). The endogenous side-product, GGOH, was also produced, along with a small quantity (~1 mg/L) of GGAc, resulting from acetylation by TAT.

Brightfield microscopy revealed no signs of filamentous growth throughout the 1L cultivations with the optimized media combination. In addition, none of the dodecanol or dodecanone compounds were detected in the solvent extracts, suggesting that the medium optimization successfully alleviated the nutritional stress and maximized taxane production by *LRS6.* However, as the observed stress response was likely multifactorial, further investigation is needed to fully characterize it and ensure the optimal performance of the engineered strain.

## 4. Conclusions

Batch-to-batch variation in the yeast extract composition was found to trigger nutritional stress in the *S. cerevisiae* strain, *LRS6.* Profound changes in the cellular morphology accompanied with biofilm formation was observed in YPG MiniBio 500 cultivations, severely hindering heterologous taxane production. Through doubling the initial yeast extract and peptone concentration (2×YP), filamentous growth was delayed and taxane accumulation improved to 108 mg/L. By coupling a statistical definitive screening design approach with the state-of-the-art, highly oxygenated microscale bioreactors, the total taxane titers were improved a further two-fold, compared to the 2×YP culture, to 229 mg/L. Filamentous growth was absent in the microscale cultivations, which confirmed that the deviations in the nutrient composition were not solely responsible for the phenomenon. The optimal conditions elucidated at microscale were validated in the 1L BIOSTAT cultivations. The production of the key Taxol precursors, T5αAc, was improved two-fold to 22 mg/L, compared to the previous maxima for the strain. The results highlighted the importance of maximizing process insight in the earliest stages of bioprocess development for effective optimization and scale-up. Although the sensitivity of the engineered strain to external processing conditions has been recognized in recent studies, here it was found to be more severe and less predictable than previously anticipated. Whilst defined media is likely to ensure more reliable taxane production, further investigation into the mechanisms behind filamentous growth are likely to prove beneficial to robust bioprocess optimization. Furthermore, as alcohols, including ethanol, the fermentation product, are major stress factors, future work should focus on minimizing their accumulation. This can be achieved through transitioning the mode of operation from batch to fed-batch through feeding the substrate at the rate of uptake, accumulation can be minimized and the overflow metabolism circumvented.

## Data Availability

Data available in supplementary information upon request.

## Supplementary Materials

The following are available online at https://www.mdpi.com/article/10.3390/microorganisms10010163/s1, Figure S1: Gas chromatographs resulting from solvent extracts taken from the 2×YP and 2×P cultivations. Figure S2: Online BioLector data. Table S1: Definitive screening experimental design used to optimize taxane production at microscale.
