# Supplementary material for: Enhancing Saccharomyces cerevisiae Taxane Biosynthesis and Overcoming Nutritional Stress-Induced Pseudohyphal Growth
Source: Microorganisms. 2022 Jan 13;10(1):163. doi: 10.3390/microorganisms10010163 (PMC8778766; doi:10.3390/microorganisms10010163)
Supplement: Supplementary file 1 [file microorganisms-10-00163-s001.zip › microorganisms-1508805-supplementary.pdf]

Enhancing *Saccharomyces cerevisiae* taxane biosynthesis and overcoming nutritional stress induced pseudohyphal growth – supplementary information

Supplementary figures

Figure S1: Gas chromatographs resulting from solvent extracts taken from the 2xYP and 2xP cultivations. A) Solvent blank. B) Extract taken from 2xYP cultivation at 142 hours. Extracts taken from the 2XP cultivation at 71 (C), 79 (D), 119.5 (E) and 142 (F) hours. 3

Figure S2: Online BioLector data. A) Biomass data for each of the 14 media combinations. B) pH values for each of the 14 media combinations. C) Dissolved oxygen values for each of the 14 media combinations. Values are mean ± standard deviation for triplicate cultivations. 4

Supplementary tables

Table S1: Definitive screening experimental design used to optimise taxane production at microscale. 2

Table S1: Definitive screening experimental design used to optimise taxane production at microscale.

Medium Number	Yeast extract (lot)	Yeast extract (g/L)	Peptone (g/L)	Uracil (mg/L)	Galactose (g/L)	Initial OD_{600}
1	one	45	45	76	45	1
2	two	5	5	0	5	0.1
3	two	25	45	0	5	1
4	one	25	5	76	45	0.1
5	two	45	25	76	5	0.1
6	one	5	25	0	45	1
7	two	5	45	38	45	0.1
8	one	45	5	38	5	1
9	two	5	5	76	25	1
10	one	45	45	0	25	0.1
11	two	45	5	0	45	0.55
12	one	5	45	76	5	0.55
13	one	25	25	38	25	0.55
14	two	25	25	38	25	0.55

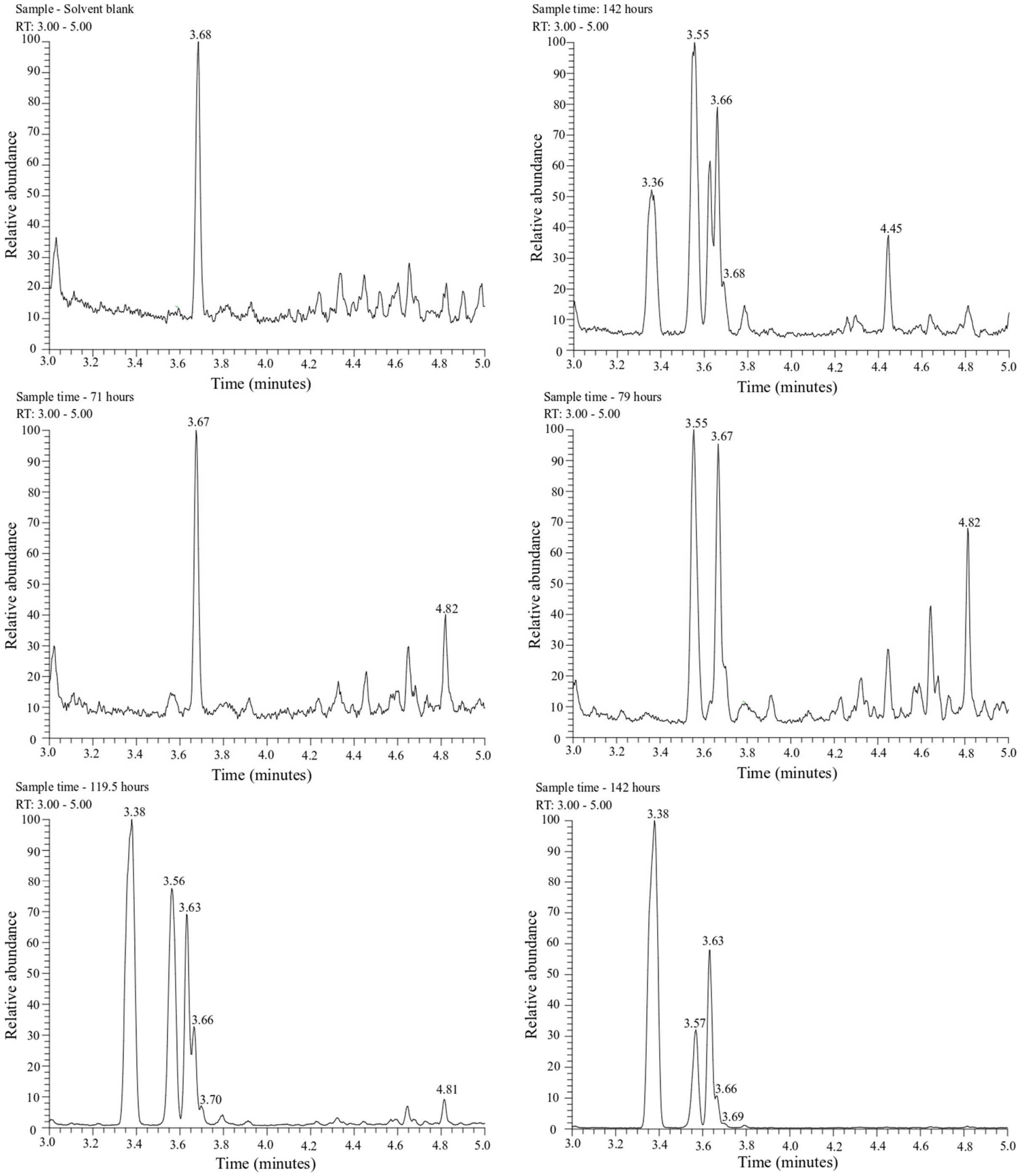

Figure S1: Gas chromatographs resulting from solvent extracts taken from the 2xYP and 2xP cultivations. A) Solvent blank. B) Extract taken from 2xYP cultivation at 142 hours. Extracts taken from the 2XP cultivation at 71 (C), 79 (D), 119.5 (E) and 142 (F) hours.

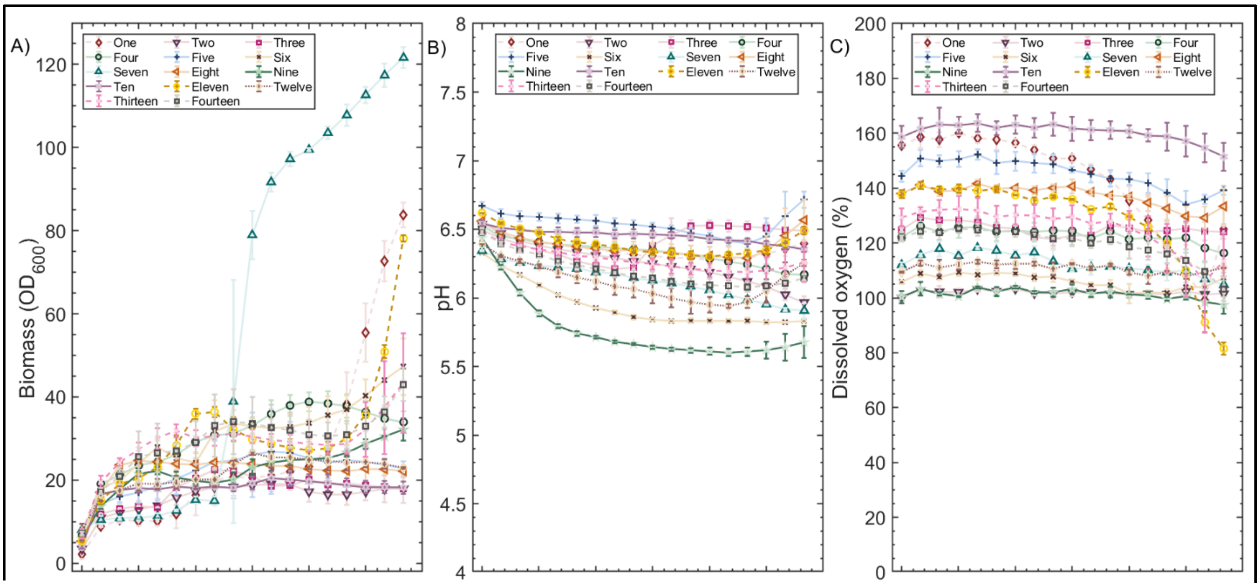

Figure S2: Online BioLector data. A) Biomass data for each of the 14 media combinations. B) pH values for each of the 14 media combinations. C) Dissolved oxygen values for each of the 14 media combinations. Values are mean ± standard deviation for triplicate cultivations.
